# Chronic Catarrh of the Stomach

**Published:** 1904-05

**Authors:** J. N. LeConte

**Affiliations:** Atlanta, Ga.


### CHRONIC CATARRH OF THE STOMACH.

BY J. N. LECONTE, M.D.,

ATLANTA, GA.

Chronic catarrh of the stomach, or chronic gastritis, includes those cases of chronic disease in which there is an inflammation of the mucous membrane, often involving the submucus and muscular layers of the stomach, which is accompanied by a disturbed secretion of gastric juice and an increased production of mucus. It is accompanied by various subjective symptoms which, however, are not characteristic of the disease.

Until recent years every case of chronic disease of the stomach which could not be diagnosed as ulcer or carcinoma, was disposed of by calling it chronic catarrh of the stomach; but modern methods of diagnosis have enabled us to define and distinguish it more accurately.

*Etiology.* Chronic gastritis begins gradually and must have some continuous or repeated causative factor that it may be developed. Among the frequent causes are: hasty eating, imperfect mastication of food, the excessive use of ice water, concentrated alcoholic drinks and tobacco, especially chewing; also the bountiful use of sauces and condiments. These, together with the nervous tension which attends our strenuous life, are all conducive to gastric catarrh, and certainly give us the right to claim dyspepsia as the national disease.

Badly decayed teeth interfere with the proper mastication of food and serve to infect it before its entrance into the stomach. Certain diseases and disturbed functions of the stomach long continued, cause gastric catarrh. It is probable that a functional hyperacidity can bring about such constant irritation of the mucous membrane that a chronic gastritis is set up, and it is reasonable to believe that in this way is caused the variety known as acid gastritis, where we have an excessive acidity with the other symptoms of chronic gastritis. Secondary catarrh may be caused by carcinoma, marked atony of the stomach, and those diseases which cause venous stasis in the stomach or affect its nutrition through the blood, such as diseases of the heart, liver, lungs, or severe forms of anemia or diabetes.

*Pathology.* The mucous membrane is generally described as being thickened, swollen, of a grayish or dark-red color and covered with a thick tenacious layer of clear or opaque mucus, all of which is more noticeable at the pyloric end.

The submucous and muscular layers often take part in the pathological changes, and upon this depends the presence or absence of sclerosis of the stomach or marked atony and dilation. The epithelium of the gastric glands undergoes fatty degeneration and the peptic can not be distinguished from the oxyntic cells, the columnar epithelium covering the mucous membrane undergoes a mucoid degeneration and subsequent desquamation. The glands are often distorted in shape from the inflammation which exists in and around them and the connective tissue becomes increased and thickened, producing pressure on the glands.

This degeneration of the secretory epithelium continuing with the hyperplasia of the connective tissue, we have as a terminal stage complete atrophy of the gastric glands, where neither ferments, acid nor mucus is produced. This gastric atrophy is generally classified as one of the forms of chronic gastritis, but, in reality, it is not gastritis at all, but the result of it.

*Symptoms.* As a rule chronic gastritis develops slowly, the symptoms at first being now present, now absent. There is usually lack of appetite, a sense of pressure and discomfort in the upper abdomen, especially after eating, but sharp pain is not frequent, unless very indigestible articles have been taken. Eructation of gas, heartburn and more or less nausea are usually seen, but vomiting is not frequent, except in the catarrh of drinkers. Weakness in the limbs, loss of energy and melancholia are often present, and in advanced cases a pronounced emaciation and anemia. Patients may appear well nourished, but in these cases the motility of the stomach is good and the intestines have performed vicariously the work of digesting the food. The so-called dyspeptic asthma, which shows itself as a shortness of breath, accompanied by palpitation, is occasionally seen, though the lungs and heart may reveal no adequate cause. Constipation is the rule in these cases, though it may alternate with diarrhea.

We gain very much more of practical knowledge from the objective than the subjective symptoms. Inspection of the abdomen reveals nothing unless there be atony or dilatation, when the epigastrium will be distended; on palpation we find marked tenderness over the stomach. Examination of the contents of the fasting stomach on rising in the morning and after an Ewald test-breakfast are all-important; indeed a positive and accurate diagnosis can not be made without repeated analyses.

On washing out the fasting-stomach in the morning, the first water often comes back perfectly clear in a way to deceive the unwary, but the use of further wash-water, especially if holding in solution bicarbonate of soda, common salt or bismuth subnitrate, detaches the thick, slimy mucus from the wall of the stomach.

Motility of the stomach, as a rule, is good, as shown by the stomach being empty of food in the morning. It is necessary to carefully differentiate stomach mucus, which is thick, glassy and tenacious, from pharyngeal mucus, which may have been swallowed, and floats on the water as opaque or yellow balls and often admixed with air-bubbles. Ewald's test-meal of bread and water being extracted after an hour's interval, is seen to consist largely of tough, thick mucus, in which are embedded the pieces of bread, swollen and unchanged, as if they had just been swallowed. On analysis we find free Hcl greatly diminished or entirely absent, combined Hcl pepsin and rennin are also more or less diminished, especially in advanced cases. Unless there is considerable atony and retention of food, the organic acids, such as acetic, lactic and butyric, are not present.

Constant presence of mucus and diminished secretion of gastric juice constitute the two most reliable symptoms in simple catarrh of the stomach.

It is often of advantage to wash out the stomach after expression of the test-meal, when we may find that a stomach which fasting was free of mucus, now contains large amounts.

Boas first described an acid gastritis, in which the symptom complex of chronic gastritis was accompanied by a normal or slightly excessive amount of free Hcl and an abundant production of mucus. It is probably most seen in the initial stage of chronic gastritis, and is considered quite rare, but I have seen a number of cases which undoubtedly belonged to this class, and in my experience is pre-eminently the form of chronic gastritis found in children. In cases where the free acid is normal or increased, the mucus is generally in shreds or fibers and has an opaque, whitish appearance; the less the percentage of acids the more swollen and glassy the mucus.

Many of these cases appear to go through a cycle, beginning with a nervous hyper-acidity, the functionating power of the stomach becomes more and more disturbed, till a chronic gastritis sets up and for a variable period of time we may find an excessive amount of free Hcl, accompanied by large quantities of opaque, thready mucus. With the advancing course of the disease, if unchecked, the free Hcl becomes less and less and finally disappears, then the combined Hcl and pepsin and rennin ferments, till finally the mucus also disappears and the case ceases to be gastritis and becomes one of gastric atrophy, where there is no gastric juice or mucus secreted in the stomach and the glands become atrophied. Though achylia gastrica, or total absence of gastric juice, may have other causes, in many cases it represents the end product of a chronic gastric catarrh. Moreover, a number of cases of chronic catarrh, applying for treatment early in the disease, having Hcl deficient or entirely absent and an abundant production of mucus, have been freed of the secretion of mucus after some weeks of continuous treatment, and hyper-acidity again showed itself. We frequently see catarrhal jaundice accompanying gastric catarrh, which is easily explained by the extension of the inflammation into the duodenum and up the gall ducts.

*Diagnosis.* The diagnosis of chronic gastritis is not so easy as the older clinicians would have us believe—the loss of appetite, distress and fullness in the epigastrium after eating, physical and mental lassitude, eructation of gas, nausea and occasional vomiting are often seen in other maladies; the chemical examination of the contents of the fasting-stomach and after a test-meal are absolutely necessary for an accurate diagnosis.

It is worth while to keep before us the dictum of Riegel, "The presence of large quantities of mucus, provided it can be demonstrated that it comes from the stomach, is an absolutely positive criterion."

This, with the decrease in the percentage of Hcl, both of which are found on repeated examinations of the stomach contents, aid us more than subjective symptoms.

Neuroses of the stomach often present many similar symptoms, but in a neurosis, these are not so constant nor so uniform nor is the Hcl so uniform in its percentages. Large amounts of stomach mucus also speak against neuroses.

Ulcer is generally accompanied by sharp, localized pain, hæmatemesis and circumscribed points of tenderness, and the vomit usually contains no mucus, but an excess of Hcl.

Carcinoma is most likely to be confounded with chronic gastric catarrh; indeed, catarrh is often found in connection with carcinoma.

A palpable tumor, more sudden onset of severe symptoms, retention of food in the stomach for an abnormal period of time, and the presence of lactic acid would lead us to suspect carcinoma.

*Prognosis.* The prognosis, as a rule, is good, but will depend upon the extent to which the secretion of gastric juice and the motor-power of the stomach is damaged; cases which are mild and not of long standing usually have a reasonably prompt recovery. Many cases which never experience a normal return of Hcl and digestive ferments, have freedom from all subjective symptoms by the aid of the motor-power of the stomach, combined with the vicarious digestive work of the small intestine.

Hemmeter has said that so long as there is any remnant of pepsin or rennin ferment, there is hope for more or less complete restitution to normal of the gastric juice. The prognosis of secondary gastric catarrh, of course, depends on the relief of the primary cause, which, in cases of venous stasis from heart or liver disease, is seldom entirely removed.

*Treatment.* Treatment may be divided into local, dietetic and medicinal.

By far the most important method of treatment is the use of lavage to free the stomach of accumulated mucus, to remove the retained decomposed food if present, and to apply medicated solutions to the wall of the stomach. It is best given in the morning before the patient has breakfasted, and may be required every other day, every day, or even twice a day on those pronounced cases which are complicated with atony and retention of food. Retained food, if present, should be first removed with plain warm water; if absent, the water often returns perfectly clear, but the use of sodium bicarbonate, calcined magnesia or common salt, will serve to dislodge the mucus which is adherent to the mucous membrane.

Several douches have been devised by means of which the fluid impinges upon the mucous membrane of the stomach through numerous small apertures, and the fluid is returned through a larger one, the reservoir being placed three feet above the cardiac opening of the stomach.

In cases complicated by atony, and having deficient Hcl, a one per cent. salt solution used in this douche is very valuable, stimulating the musculature and secretion of Hcl. In cases of acid catarrh and those having erosions, I have seen very gratifying results from the use of a solution of silver nitrate, one to two thousand, in this douche, always being careful to remove all the solution, or neutralize afterward with sodium chloride.

Disinfectants, such as solutions of boric acid or permanganate of potash, are useful in cases of fermentation.

Intragastric faradic electricity is of value (after the large amounts of mucus have been checked by methodic lavage) to stimulate an increased amount of Hcl and restore tone to the mucosa and muscularis.

The galvanic current may be used where the patient complains of much pain.

The quantity and quality of the food will depend upon the state of the secretion in the stomach and its motor-power, but it is not wise to direct too strict a diet, especially in advanced cases, where maintenance of the nutrition is all-important. Albumenoids, starches and fats should all be represented in the diet. All food, especially albumenoids, should be served in a soft, finely divided state; vegetables or meats containing hard or tough fibers should be rejected, or any food having an irritating effect.

It is often well to direct five or six small meals a day. Concentrated alcoholics should be absolutely interdicted. Of late years drugs are used much less than formerly in the treatment of chronic gastritis. Diluted hydrochloric acid may be used to advantage where the natural acid is shown to be deficient; however, a certain percentage of these cases of long standing are peculiarly intolerant of hydrochloric acid. Strychnia, in the form of nux-vomica, or some other stomachic bitter, such as condurango or quassia, administered before meals, do much to spur up the jaded appetite and thereby improve nutrition. The constipation which is so frequent a symptom must be combated by a well selected diet, regular habits, the use of a glass of cold water containing a pinch of salt on rising, or, if necessary, a half pint of olive oil as an enema. Drug laxatives are distinctly harmful and should be used as little as possible. In cases of fermentation a combination of thymol with boric acid may be given to advantage.

Lastly the patient should be advised as to those hygienic common-sense laws which are conducive to recovery in any chronic digestive disturbance. Food should be masticated slowly and thoroughly, and if the teeth are very defective, artificial ones should be fitted. Gentle outdoor exercise, proper ventilation of sleeping apartments, the avoidance of severe mental or physical strain, the use of a cold sponge-bath and a brisk rub-down in the morning, the use of a limited amount of fluid at meals and a reasonable quantity between—all of these should be considered in giving directions to the patient.

